# Digital Biomarkers of Gait and Balance in Diabetic Foot, Measurable by Wearable Inertial Measurement Units: A Mini Review

**DOI:** 10.3390/s22239278

**Published:** 2022-11-29

**Authors:** Gu Eon Kang, Angeloh Stout, Ke’Vaughn Waldon, Seungmin Kang, Amanda L. Killeen, Peter A. Crisologo, Michael Siah, Daniel Jupiter, Bijan Najafi, Ashkan Vaziri, Lawrence A. Lavery

**Affiliations:** 1 Department of Bioengineering, Erik Jonsson School of Engineering & Computer Science, The University of Texas at Dallas, Richardson, TX 75080, USA; 2 Department of Plastic Surgery, The University of Texas Southwestern Medical Center, Dallas, TX 75390, USA; 3 Department of Surgery, The University of Texas Southwestern Medical Center, Dallas, TX 75390, USA; 4 Department of Biostatistics and Data Science, Department of Orthopaedic Surgery and Rehabilitation, The University of Texas Medical Branch at Galveston, Galveston, TX 77555, USA; 5 Michael E. DeBakey Department of Surgery, Baylor College of Medicine, Houston, TX 77030, USA; 6 BioSensics LLC, Newton, MA 02458, USA

**Keywords:** diabetic foot, diabetic neuropathies, peripheral arterial disease, foot ulcer, gait, walking, postural balance, wearable electronic devices, inertial measurement unit, digital technology

## Abstract

People with diabetic foot frequently exhibit gait and balance dysfunction. Recent advances in wearable inertial measurement units (IMUs) enable to assess some of the gait and balance dysfunction associated with diabetic foot (i.e., digital biomarkers of gait and balance). However, there is no review to inform digital biomarkers of gait and balance dysfunction related to diabetic foot, measurable by wearable IMUs (e.g., what gait and balance parameters can wearable IMUs collect? Are the measurements repeatable?). Accordingly, we conducted a web-based, mini review using PubMed. Our search was limited to human subjects and English-written papers published in peer-reviewed journals. We identified 20 papers in this mini review. We found preliminary evidence of digital biomarkers of gait and balance dysfunction in people with diabetic foot, such as slow gait speed, large gait variability, unstable gait initiation, and large body sway. However, due to heterogeneities in included papers in terms of study design, movement tasks, and small sample size, more studies are recommended to confirm this preliminary evidence. Additionally, based on our mini review, we recommend establishing appropriate strategies to successfully incorporate wearable-based assessment into clinical practice for diabetic foot care.

## 1. Introduction

The global epidemic of diabetes imposes significant burdens on healthcare systems [1]. The International Diabetes Federation estimated that in the year 2021, 537 million people were living with diabetes worldwide, and that this number would increase to 643 million by the year 2030 and 783 million by the year 2045 [2]. Diabetes is a cause of 6.7 million deaths worldwide [2]. Medical expenditures for diabetes are enormous: more than $200 billion in the USA alone and nearly $1 trillion worldwide [2,3].

Diabetes comprises a group of disorders that results in high blood-glucose levels, namely hyperglycemia, caused by deficits in insulin response [4]. There are two main types of diabetes: type 1 diabetes, also known as insulin-dependent diabetes, and type 2 diabetes, characterized by insulin-resistance [5,6]. More than 90% of people with diabetes have type 2 diabetes [6]. If poorly managed, diabetes causes a number of complications.

Diabetic foot, defined as “infection, ulceration or destruction of tissues of the foot associated with diabetic neuropathy or peripheral artery disease in the lower limb of a person with diabetes” [7], is one of the most common and devastating complications of diabetes, which affects 2–6% of people with diabetes annually [8,9]. Diabetic foot accounts for more than 100,000 lower-extremity amputations in the USA and more than 1,000,000 lower-extremity amputations worldwide each year [10,11]. Remarkably, lower-extremity amputation is more fearful than death for people with diabetic foot [12].

Although diabetic foot is generally a consequence of multiple factors, common causal factors are sensory neuropathy causing sensory loss, motor neuropathy causing biomechanical abnormalities, autonomic neuropathy causing dry skin, and peripheral arterial disease causing claudication, rest pain, and tissue loss in the lower-extremity [13]. All these factors inherently limit gait and balance [14].

Gait and balance dysfunction has significant negative impacts on survival and quality of life of people with diabetic foot [15]. For example, the risk of fall and likelihood to be injured from a fall in people with diabetic foot is 23 and 15 times greater, respectively, than in people without diabetic foot [16]. Gait and balance dysfunction in people with diabetic foot is associated with an onset or progression of fear of falling, and restricts normal daily activities [17,18,19]. Furthermore, gait and balance dysfunction alters kinematics and kinetics, such as excessive plantar pressure and shear stress, and may contribute to the development of a foot ulcer or leads to deterioration of an already developed foot ulcer [20,21]. Thus, assessing gait and balance with valid and effective tools has been a critically important aspect of the management of diabetic foot.

Traditionally, visual observation in clinical settings or 3D-optoelectronic-motion capture systems in laboratory settings have been popularly utilized to assess gait and balance in people with diabetic foot. However, visual observation relies on clinicians’ experiences and is limited to gait speed, and 3D-optoelectronic-motion capture systems are expensive, time-consuming, and may not be suitable for translational research [22,23]. Instrumented walkways are another popular method; however, they are also expensive and limited to a relatively short distance (four to eight meters).

Wearable inertial measurement units (IMUs) are a viable option that can address limitations of the popular methods. Wearable IMUs, typically composed of an accelerometer and a gyroscope, have shown to provide repeatable and valid data in gait and balance assessment across clinical settings [24,25]. For example, Schwenk and colleagues used five IMUs attached on the shins, thighs, and lower back, and reported spatiotemporal parameters during gait and balance parameters during quiet standing tasks [26]. Furthermore, recent technical advances enabled wearable IMUs to assess gait and balance outside traditional gait laboratories in diabetic foot [27].

Nevertheless, we found no published reviews regarding these aspects. Accordingly, we aimed to summarize up-to-date findings regarding gait and balance assessment in people with diabetic foot using wearable IMUs. In particular, because gait and balance assessment using wearable IMUs is an emerging area of research, which has been studied only in recent years, and our topics (i.e., diabetic foot, wearable IMUs, gait and balance) are specific rather than broad, we conducted a mini review in this study. The primary focuses of our mini review were parameters of gait and balance measurable by wearable IMUs in people with diabetic foot, and repeatability of such parameters. Additionally, based on our mini review, we aimed to discuss limitations from previous papers and suggest areas of future research in gait and balance assessment in people with diabetic foot using IMUs.

## 2. Materials and Methods

Since this study was a mini review, we performed a web-based, electronic search using one database, PubMed (which covers a significant number of citations [≥34 million] for biomedical research papers), for papers published before 9 August 2022. The following terms were used for PubMed search: “diabetic neuropathies”, “diabetic foot”, “peripheral arterial disease”, “foot ulcer”, “gait”, “walking”, and “postural balance”. The full search query is described in Table 1.

Our inclusion criteria were English-written papers that assessed gait and balance performance using IMUs in people with diabetic foot. If a paper utilized either an accelerometer or a gyroscope, we included the paper. Additional inclusion criteria were papers reported outcomes related to kinematic variables during gait and/or quiet standing, such as spatiotemporal parameters during gait or center-of-mass displacement during quiet standing. An experienced reviewer (G.E.K.) conducted screening of the searched papers based on titles and abstracts. This reviewer had sufficient experiences in such tasks and published multiple reviews previously [28,29].

Exclusion criteria were review papers, editorial comments, conference abstracts, and letters from the final paper selection. We limited our search of papers to human-subject studies, and thus, excluded animal studies. Additionally, if a paper reported a validity of IMUs in comparison to another motion-analysis system or subjective reports within one group, we excluded the paper. Furthermore, because the focus of this review is diabetic foot, if a paper included people with diabetes, assessed gait, and balance performance using IMUs, but did not specify the presence or a diagnosis of foot complications in the people of diabetes, the paper was excluded from final selection.

## 3. Results

### 3.1. Search Results

**Figure 1:**
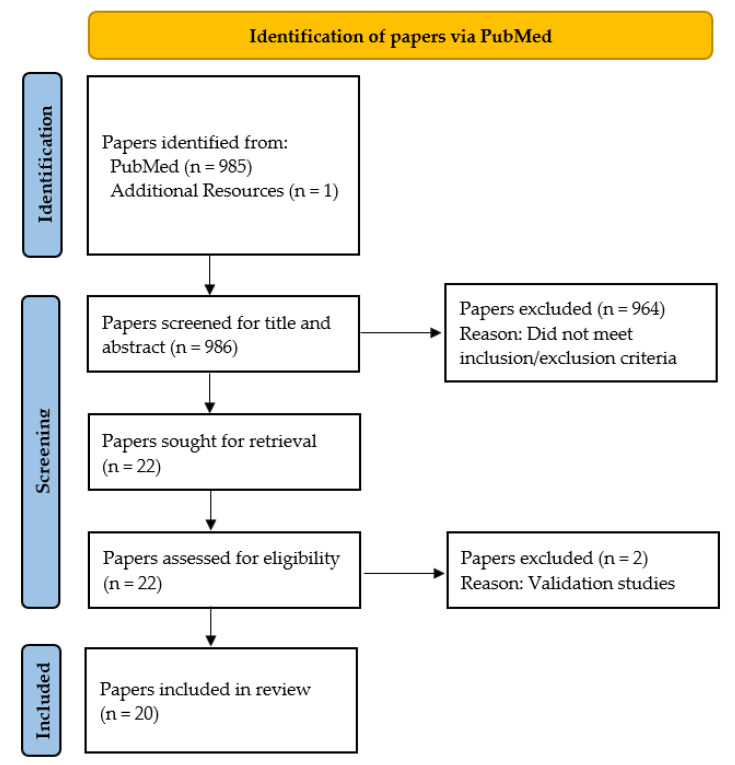
Flow chart for selecting papers.

The flow diagram for paper selection is shown in Figure 1. A total of 986 papers were identified through PubMed. After screening titles and abstracts, 964 papers were excluded because they did not meet our inclusion and exclusion criteria, described in the previous section. After evaluating eligibility of the remaining 22 articles, two more papers were excluded based on full-text review because they were validation studies comparing two different measurements. Consequently, 20 papers were included in the current review [30,31,32,33,34,35,36,37,38,39,40,41,42,43,44,45,46,47,48,49].

### 3.2. Study Characteristics

We summarized the findings from the final 20 papers in Table 2. All included papers were published between 2004 and 2021: a total of thirteen studies were conducted in the USA, three studies in Switzerland, one study in Australia, one study in the UK, one study in China, and one study jointly conducted in the USA and Qatar.

### 3.3. Study Design and Participant Characteristics

Of the twenty included studies, seventeen studies included people with diabetic-peripheral neuropathy, and three studies included people with peripheral-arterial disease. A total of thirteen studies were non-interventional observational studies, and seven studies were interventional studies, among which four studies were randomized controlled trials.

Among the thirteen observational studies, eight studies compared people with diabetic-peripheral neuropathy and healthy controls, among which two studies included those with active diabetic-foot ulcer; and one study compared people with peripheral- artery disease and healthy controls. Three studies included only one group of people with diabetic-peripheral neuropathy, and one study included only one group of people with recently healed diabetic-foot ulcer.

Four randomized controlled trials tested the effectiveness of exercise or electrical stimulation within groups of people with diabetic-peripheral neuropathy. Three non-randomized interventional studies tested the effectiveness of diabetic-foot orthoses and mechanical stimulation in people with diabetic-peripheral neuropathy, and the effectiveness of exercise in people with peripheral-artery disease.

For the seventeen studies which included people with diabetic-peripheral neuropathy, eleven studies reported vibration-perception threshold to measure the severity of neuropathy, and six studies reported blood sugar level. The three studies which included people with peripheral-artery disease, reported ankle-brachial index to assess lower-extremity blood flow.

### 3.4. Tasks and IMUs

**Figure 2:**
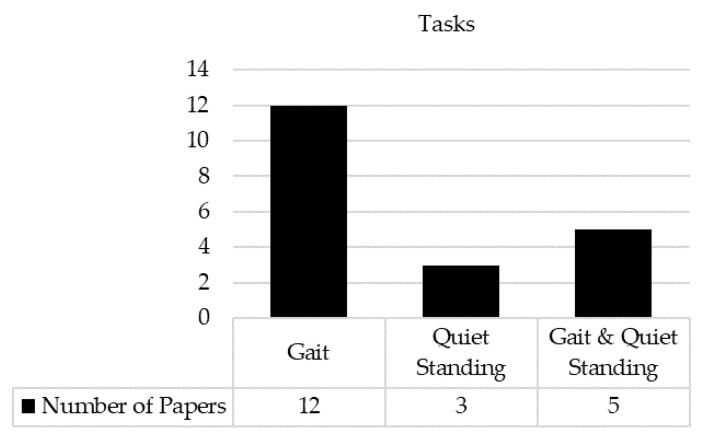
The number of papers for each task.

Common tasks assessed with IMUs were gait and quiet standing in various conditions. Of the twenty studies included, twelve studies tested gait, three studies tested quiet standing, and the other five studies tested both gait and quiet standing (Figure 2).

**Figure 3:**
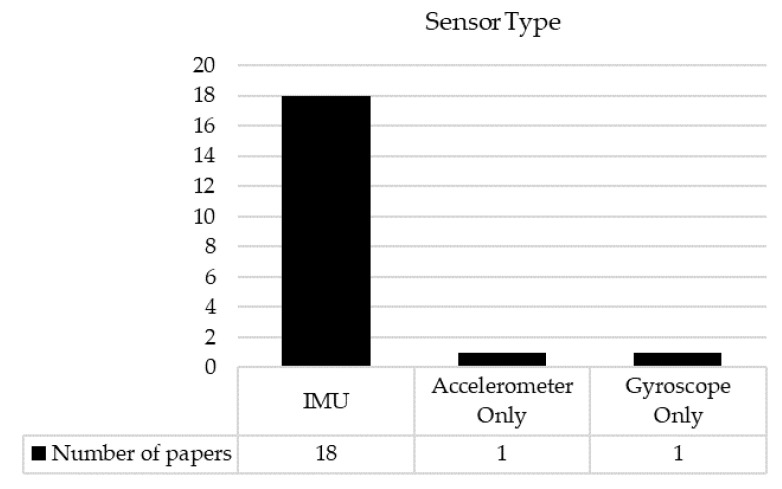
The number of papers for each sensor type.

In terms of methods, of the twenty included studies, eighteen studies used IMUs and two studies used either 3D accelerometers or gyroscopes (Figure 3). The number of sensors was between one and five. Sampling frequencies, if reported, were either 100 Hz or 200 Hz. Common sensor positions for gait assessment were the lower back, thighs, and shins for the five-sensor system; the thighs and shins for the four-sensor system; the shins for the two-sensor system; and the lower back for the one-sensor system. One study that used 3D accelerometers for gait assessment attached the sensors to the head and lower back. For assessing balance during quiet standing, two IMUs were commonly used with the sensors attached to the lower back and shin.

### 3.5. Measures and Key Findings

**Figure 4:**
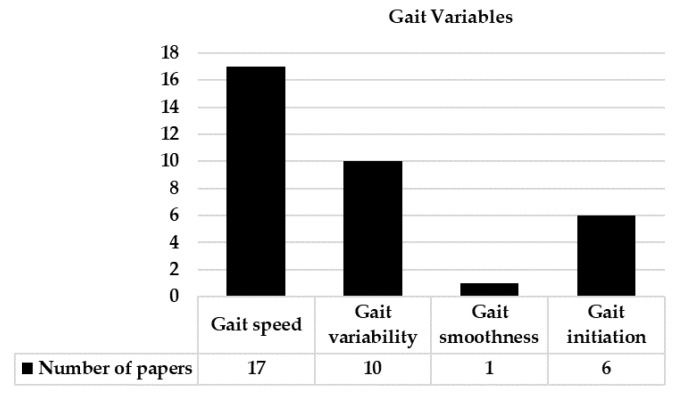
The number of papers for each gait variable.

The most popular gait measures were gait speed, stride length (or step length), and stride time (or gait cycle time or step time), each of which was reported in 100% of gait studies (Figure 4). Gait-variability measures (i.e., fluctuations in stride-to-stride), such as gait-speed variability, stride-time variability, and stride-length variability were reported in ten studies. Gait-initiation variables, including the number of steps and distance to be taken from standing posture to steady state walking, were reported in six studies. There was one study which quantified smoothness using the harmonic ratio, and another study quantified limping during gait. Besides, one study reported foot-kinematic variables, such as toe clearance.

**Figure 5:**
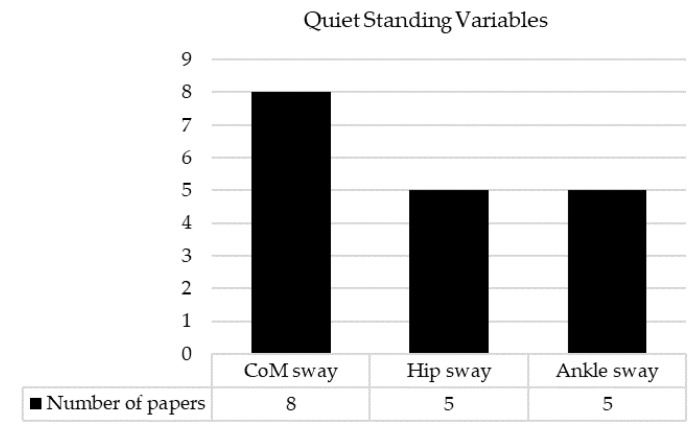
The number of papers for each quiet standing variable.

In terms of quiet standing, center-of-mass sway was the most popular measure, which was reported in 100% of quiet-standing studies (Figure 5). Other variables of quiet standing included ankle sway and hip sway. There was one study which reported local- control balance and central-control balance.

In terms of key findings, gait studies reported slow gait speed, shorter stride length, greater gait variability, and longer gait-initiation phase in people with diabetic foot, compared to control subjects (e.g., healthy controls, people with diabetes but no diabetic foot). Similarly, studies that measured quiet standing reported larger sway in center-of-mass, ankle, or hip in people with diabetic foot, compared to control subjects. Interventional studies also reported improvements in these gait and quiet-standing measures at post-intervention, compared to pre-intervention.

## 4. Discussion

### 4.1. Summary

We aimed to provide an up-to-date review of the existing literature regarding assessment of gait and balance using wearable IMUs in people with diabetic foot. Given the main role of the foot during gait (i.e., force absorption) and biomechanical deformities in diabetic foot, the importance of assessing gait and balance has been continuously emphasized in numerous review papers [14,15,50,51]. Furthermore, gait and balance dysfunction is the key indicator of increased risk of falling in people with diabetic foot, which might in turn increase risk of hospitalization [52,53]. Gait and balance dysfunction may facilitate ulceration because of abnormal loading pattern [54].

In this mini review, we identified a total of 20 papers that met our inclusion and exclusion criteria. Although there were some heterogeneities in gait and quiet-standing protocols and findings, across the reviewed studies, key parameters of IMU-based gait and balance assessment were gait speed, gait-initiation steps and distance, gait variability, and body sway during quiet standing. Furthermore, reviewed studies demonstrated reasonably consistent patterns of such parameters in people with diabetic foot in comparison to non-diabetic people or in response to an intervention.

Based on these findings, our mini review suggests IMUs may have potential to be used in clinical settings to measure kinematic aspects of gait and balance dysfunction in people with diabetic-peripheral neuropathy, active diabetic-foot ulcer, or peripheral- artery disease. Furthermore, IMU-based parameters could assist in designing remote-patient-monitoring platform to track changes in digital biomarkers of gait and balance dysfunction among people with diabetic foot. To our knowledge, this is the first review (regardless of the type of the review) that focused on gait and balance assessment using IMUs in people with diabetic foot. Our review may also be used as the first step towards establishing a general agreement on gait metrics specifically described for people with diabetic foot.

### 4.2. Challenges and Future Directions

From this review, we realized an agreed protocol for IMU-based gait assessment is urgently needed. Although most of the studies that tested IMU-based quiet standing used similar protocols adopted from the Romberg test [55], protocols for gait assessment varied significantly between studies in terms of distance, single or dual task conditions, speed conditions, and footwear conditions. These varied conditions might have resulted in heterogeneities in gait results. Furthermore, IMU-based gait outcomes that can indicate an important sub-phase of gait cycle, such as propulsion phase or breaking phase, would be beneficial. Because these phases account for the greatest shear and vertical pressure on the foot during gait, which is directly associated with an onset of an ulcer (i.e., skin breakdown) or progressing ulcerations, a way to assess characteristics of these phases will be particularly beneficial for people with diabetic foot.

Another important issue is the repeatability. Repeatability of IMU-based, gait analysis and balance assessment has been reported in general or other clinical populations [56,57]. For example, Washabaugh and colleagues used commercially available IMUs (APDM Inc., Portland, OR; *n* = 2), attached on the feet or ankles, and evaluated repeatability of the IMUs in measuring spatiotemporal-gait parameters during overground gait (three trials; healthy young adults), including gait speed, stride length, and cadence [56]. They reported high repeatability of the IMU-based, spatiotemporal-gait parameters. Felius and colleagues used a commercially available IMU (Aemics b.v. Olden-zaal, The Netherlands) and evaluated repeatability of the IMUs in measuring balance parameters of the trunk in quiet standing [57]. They reported medium-to-high repeatability of the IMU-based balance parameters. However, we were unable to find evidence of repeatability in people with diabetic foot. It is urgently needed to investigate if IMUs can provide repeatable gait or balance parameters in diabetic foot.

Small sample sizes in nearly every included study is another issue. Surprisingly, regardless of study design, the maximum number of participants in a group (either intervention group or control group in a randomized controlled trial) was 38. These two issues can lead to a subsequent question of the generalizability of the findings. In terms of participant characteristics included in this review, the vast majority of the recruited people had diabetic-peripheral neuropathy, the most common underlying etiology causing a diabetic foot ulcer [58], but reports are needed for different diabetic-foot problems, such as Charcot foot and diabetic foot in remission [59,60].

Limitations of this review should be acknowledged. Due to the issues regarding study protocol, repeatability, and small sample size, we recommend considering our review as a preliminary review, not a confirmatory work. Another limitation is heterogeneity in IMUs. Although validations of the IMUs that were chosen in each paper have been reported previously, different IMUs may have slightly different results for sampling frequency and filtering techniques, though this has not been reported in the included papers.

Despite these limitations, based on the current status of using IMUs in assessing gait and balance in the management of diabetic foot, we believe the following examples are areas of future research. One primary area is to establish an implementation strategy. One strength of IMUs is the possibility of them being incorporated into clinical practice. IMUs provide more detailed and necessary information about a person’s functional status comparable to a stopwatch, and are more portable and translational, compared to three-dimensional, optoelectronic-motion-capture technology. In fact, the importance of implementation has been discussed previously [27,61], and implementation has been attempted in another population [62]. We believe appropriate strategies, such as IMU-based, perioperative-gait assessment will significantly advance the management of diabetic foot. Another primary area is to better identify people at the highest risk of diabetic-foot ulceration. This may be particularly beneficial for those who have recently recovered from a diabetic-foot ulcer. IMU-based gait assessment during clinic visits on a regular basis (e.g., every three or six months according to established guidelines) may better identify those whose ulcers are likely to recur and those who will likely remain ulcer-free.

## 5. Conclusions

Assessing gait and balance dysfunction and investigating biomechanics have undoubtedly advanced our understanding of diabetic-foot syndrome. Based on our review, we found that IMU-based gait and balance assessment can provide information regarding gait analysis, gait initiation, and gait variability, and body sway during quiet standing. Our review identified several issues and limitations of the included studies, and suggested future directions that may address current limitations and achieve advancements in diabetic-foot management. We believe rapid developments in sensing technology and data-analysis technology will further speed up the processes to successfully incorporate IMUs into clinical practice.

## Figures and Tables

**Table 1:** PubMed search query.

	Concept	Search Query
	Diabetic Foot	“Diabetic Neuropathies” [MeSH Terms] OR “Diabetic Foot” [MeSH Terms] OR “Foot Ulcer” [MeSH Terms] OR “Peripheral Arterial Disease” [MeSH Terms])
AND	Gait and Balance	“Gait” [MeSH Terms] OR “Walking” [MeSH Terms] OR “Postural Balance” [MeSH Terms])

**Table 2:** Summary of included papers. A list of abbreviations was added at the bottom of this table.

Study Title Country	Study Design Participants	Tasks	Sensor Type (Manufacturer) Placement Sampling Frequency	Key Measures	Findings
Menz et al., 2004 [30] Walking stability and sensorimotor function in older people with diabetic peripheral neuropathy Australia	Observational DPN *n* = 30 (22 men; 8 women) Age (years) = 73.5 ± 8.3 BMI (kg/m^2^) = 28.2 ± 6.0 Duration of DM (years) = 12.3 ± 8.4 HbA1c (%) = 7.6 ± 1.3 VPT (Volts) = 37.6 ± 11.4 HC *n* = 30 (22 men; 8 women) Age (years) = 73.9 ± 9.0 BMI (kg/m^2^) = 25.6 ± 3.4	Gait 20 m Two surface conditions Level Irregular	3D Accelerometer (NA) *n* = 2 Head (*n* = 1) Sacrum (*n* = 1) Frequency not reported	Gait speed Cadence Step length Step time variability Smoothness (harmonic ratio)	↓ Gait speed, cadence, step length, and smoothness on both surfaces in DPN vs. HC ↑ Step time variability on irregular surface in DPN vs. HC
Allet et al., 2009 [31] Gait alterations of diabetic patients while walking on different surfaces Switzerland	Observational DPN *n* = 15 (sex ratio not reported) Age (years) = 61.29 ± 6.52 Height (m) = 1.67 ± 0.08 Weight (kg) = 86.94 ± 9.13 Duration of DM (years) = 8.83 ± 4.60 Blood sugar level not reported VPT (Scale) = 2.63 ± 1.58 DM (without neuropathy) *n* = 15 (sex ratio not reported) Age (years) = 55.83 ± 8.20 Height (m) = 1.72 ± 0.12 Weight (kg) = 90.30 ± 22.15 Duration of DM (years) = 9.87 ± 7.78 Blood sugar level not reported VPT (Scale) = 5.65 ± 1.14 HC *n* = 15 (sex ratio not reported) Age (years) = 57.42 ± 4.31 Height (m) = 1.73 ± 0.10 Weight (kg) = 79.93 ± 11.53 VPT (Scale) = 6.80 ± 0.86	Gait Distance not reported Three surface conditions Tar Grass Stones	IMU (BioAGM, Lausanne, Switzerland) *n* = 4 Shin (*n* = 2; right and left) Thigh (*n* = 2; right and left) Frequency = 200 Hz	Gait speed Cadence Stride length Stance phase Double support Gait cycle time Step time variability	↓ Gait speed, cadence, and stride length in DPN vs. HC, but not DPN vs. DM ↑ Stance phase, double support, gait cycle time, and stride time variability on all surfaces in DPN vs. HC, but not DPN vs. DM
Allet et al., 2010 [32] An exercise intervention to improve diabetic patients’ gait in a real-life environment Switzerland	Interventional (RCT): IG (Exercise intervention 60 min per session; two sessions per week; 12 weeks) DPN *n* = 35 (sex ratio not reported) Age (years) = 63.0 ± 8.0 BMI (kg/m^2^) = 30.5 ± 6.0 Disease duration not reported Blood sugar level not reported VPT (Scale) = 3.2 ± 1.3 CG (No treatment or advice) DPN *n* = 36 (sex ratio not reported) Age (years) = 64.0 ± 8.9 BMI (kg/m^2^) = 31.5 ± 5.3 Disease duration not reported Blood sugar level not reported VPT (Scale) = 3.3 ± 1.3	Gait Distance not reported Two surface conditions Tar Cobblestone	Gyroscope (NA) *n* = 4 Shin (*n* = 2; right and left) Thigh (*n* = 2; right and left) Frequency = 200 Hz	Gait speed Cadence Stride length Stance time Gait cycle time Step time variability Time points: Baseline 12-week 6-month	All gait and balance variables were similar between IG and CG at baseline ↑ Gait speed, cadence, and stride length in IG at 12-week and 6-month vs. baseline on both surfaces ↓ Gait cycle time and stance time in IG at 12-week and 6-month vs. baseline on both surfaces
Crews et al., 2012 [33] Impact of strut height on offloading capacity of removable cast walkers USA	Observational DPN with diabetic foot risk classification: Grade 1 (*n* = 8) Grade 3 (*n* = 1) Grade 4 (*n* = 2) *n* = 11 (7 men, 4 women) Age (years) = 51.4 ± 10.0 BMI (kg/m^2^) = 33.9 ± 7.3 Duration of DM (years) = 14.5 ± 9 Blood sugar level not reported VPT value not provided	Gait 20 m Four shoe conditions Ankle-high RCW Knee-high RCW Shoe RCW Standard athletic shoe	IMU (BioAGM, Lausanne, Switzerland) *n* = 5 Shin (*n* = 2; right and left) Thigh (*n* = 2; right and left) Lumbar region (*n* = 1) Frequency = 200 Hz	Gait speed Stride length Stride time Double support Gait speed variability	↓ Gait speed and stride length in ankle-high RCW and knee-high RCW vs. standard athletic shoe ↑ Stride time, double support, and gait speed variability in ankle-high RCW and knee-high RCW vs. standard athletic shoe
Najafi et al., 2013a [34] The impact of footwear and walking distance on gait stability in diabetic patients with peripheral neuropathy USA	Observational DPN *n* = 12 (8 men; 4 women) Age (years) = 60 ± 12 BMI (kg/m^2^) = 33.2 ± 6.4 Duration of DM (years) = 10 ± 13 Blood sugar level not reported VPT (Volts; right foot) = 56 ± 25 VPT (Volts; left foot) = 61 ± 29 HC *n* = 8 (6 men; 2 women) Age (years) = 60 ± 6 BMI (kg/m^2^) = 27.0 ± 3.2 VPT (Volts; right foot) = 19 ± 4 VPT (Volts; left foot) = 20 ± 3	Gait Four conditions (two distance × two footwear) Short (7 m) Long (20 m) Barefoot Regular shoes	IMU (BioSensics, Newton, MA, USA) *n* = 5 Shin (*n* = 2; right and left) Thigh (*n* = 2; right and left) Lower back (*n* = 1) Frequency = 100 Hz	Gait initiation steps Gait initiation speed Gait speed Stride length Stride time Double limb support Gait speed variability CoM sway	All variables were similar between DPN and HC in the short-distance condition regardless of footwear conditions ↓ Gait initiation speed, gait speed, and stride length in DPN vs. HC in the long-distance condition regardless of footwear conditions ↑ Gait initiation steps, stride time, double-limb support, and gait-speed variability in the short-distance condition regardless of footwear conditions CoM sway was similar between DPN and HC in all conditions
Najafi et al., 2013b [35] A novel plantar stimulation technology for improving protective sensation and postural control in patients with diabetic peripheral neuropathy: A double-blinded, randomized study USA	Interventional (RCT) IG (Electrical plantar stimulation; 30 min per treatment; 5 treatments per week; 6 weeks) DPN *n* = 25 (sex ratio not reported) Age (years) = 61.6 ± 8.3 BMI not reported Disease duration not reported HbA1c (%) = 7.6 ± 1.6 VPT (Volts) = 46.8 ± 23 CG (Sham stimulation) DPN *n* = 29 (sex ratio not reported) Age (years) = 61.4 ± 8.2 BMI not reported Disease duration not reported HbA1c (%) = 7.1 ± 1.5 VPT (Volts) = 37.6 ± 22	Quiet standing Two conditions Eyes open Eyes closed Measured in a sub-sample	IMU (BioSensics, Newton, MA, USA) *n* = 2 Shin (*n* = 1) Lower back (*n* = 1) Frequency not reported	CoM sway area Time points: Baseline 2-week 4-week 6-week 6-month	All variables were similar between IG and CG at baseline. ↓ CoM sway area at weeks 2, 4, and 6 vs. baseline in IG ↑ CoM sway area at weeks 2, 4, and 6 vs. baseline in CG
Grewal et al., 2013 [36] Diabetic peripheral neuropathy and gait: Does footwear modify this association? USA	Observational DPN with active DFU *n* = 16 (sex ratio not reported) Age (years) = 58.3 ± 4.4 BMI (kg/m^2^) = 29.5 ± 3.7 Disease duration not reported Blood sugar level not reported VPT value not provided DPN without active DFU *n* = 15 (sex ratio not reported) Age (years) = 54.2 ± 11.3 BMI (kg/m^2^) = 31.2 ± 5.9 Disease duration not reported Blood sugar level not reported VPT value not provided HC *n* = 8 (sex ratio not reported) Age (years) = 59.6 ± 6 BMI (kg/m^2^) = 27 ± 3.2	Gait 200 feet Habitual pace	IMU (BioSensics, Newton, MA, USA) Sensor placement not reported Sampling frequency not reported	Gait initiation steps Gait initiation distance Gait speed Stride length Gait cycle time Double stance Gait speed variability CoM sway Knee RoM	↑ Gait initiation steps, gait speed variability in DPN groups vs. HC ↓ Knee RoM in DPN groups vs. HC
Kelly et al., 2013 [37] Fear of falling is prevalent in older adults with diabetes mellitus but is unrelated to level of neuropathy USA	Observational DPN *n* = 16 (10 men; 6 women) Age (years) = 73 ± 8 BMI (kg/m^2^) = 30.6 ± 5.7 Duration of DM (years) = 17 ± 11 HbA1c (%) = 8.9 ± 2.7 VPT (Volts) = 49.7 ± 21.9 DM without neuropathy *n* = 18 (5 men; 13 women) Age (years) = 62 ± 7 BMI (kg/m^2^) = 31.2 ± 5.9 Duration of DM (years) = 13 ± 13 HbA1c (%) = 7.2 ± 1.6 VPT (Volts) = 18.3 ± 4.5	Gait 20 m Habitual pace	IMU (BioSensics, Newton, MA, USA) *n* = 5 Shin (*n* = 2; right and left) Thigh (*n* = 2; right and left) Lower back (*n* = 1) Frequency not reported	Gait initiation steps Gait speed Stride length Stride time Double stance Gait speed variability CoM sway	↑ Gait initiation steps and double stance in DPN vs. DM without neuropathy Gait initiation steps and double stance were significantly correlated with VPT
Wrobel et al., 2014 [38] A novel shear reduction insole effect on the thermal response to walking stress, balance, and gait for diabetic neuropathy USA	Interventional DFO; immediate effect DPN *n* = 27 (14 men; 13 women) Age (years) = 65.1 BMI (kg/m^2^) = 33.9 Disease duration not reported Blood sugar level not reported VPT value not provided	Gait 200 steps Two conditions Habitual pace Dual task Quiet standing Two conditions Eyes open Eyes closed	IMU (BioSensics, Newton, MA, USA) Sensor placement not reported Frequency not reported	Gait Gait initiation steps Gait initiation speed Gait initiation double stance Gait speed Stride length Stride time Double stance Gait speed variability CoM sway Quiet standing CoM sway area	↓ Gait initiation double stance for DFO vs. standard shoe during habitual walking
Grewal et al., 2015 [39] Sensor-based interactive balance training with visual joint movement feedback for improving postural stability in diabetics with peripheral neuropathy: A randomized controlled trial USA	Interventional (RCT) IG (Balance training exercise with real-time visual feedback; twice a week; 4 weeks) DPN *n* = 19 (male = 8, female = 11) Age (years) = 62.58 ± 7.98 BMI (kg/m^2^) = 31.78 ± 7.53 Duration of DM (years) = 17.17 ± 10.08 HbA1c (mmol/mol) = 65.23 ± 19.65 VPT (Volts) = 34.28 ± 8.16 CG (Not specified) DPN *n* = 16 (male = 8, female = 8) Age (years) = 64.90 ± 8.50 BMI (kg/m^2^) = 29.58 ± 4.24 Duration of DM (years) = 17.40 ± 9.42 HbA1c (mmol/mol) = 65.40 ± 29.91 VPT (V) = 33.52 ± 6.16	Quiet standing Two condition Eyes open Eyes closed	IMU (BioSensics, Newton, MA, USA) *n* = 2 Shin (*n* = 1) Lower back (*n* = 1) Frequency = 100 Hz	CoM sway Ankle sway Hip sway Time points: Baseline 4-week	↓ CoM, ankle and hip sway at 4-week vs. baseline in IG during eyes open
Toosizadeh et al., 2015 [40] The influence of diabetic-peripheral neuropathy on local postural muscle and central sensory feedback balance control USA	Observational DPN *n* = 18 (11 men; 7 women) Age (years) = 65 ± 8 BMI (kg/m^2^) = 29.3 ± 5.4 Duration of DM (years) = 19 ± 11 Blood sugar level not reported VPT (mV) = 34.6 ± 7.0 HC *n* = 18 (7 men; 11 women) Age (years) = 69 ± 3 BMI (kg/m^2^) = 27.0 ± 4.1	Quiet standing Two conditions Eyes open Eyes closed	IMU (BioSensics, Newton, MA, USA) *n* = 2 Sensor placement not reported Frequency not reported	CoM sway Local-control balance Central-control balance	↑ CoM sway, local-control balance, and central-control balance in DPN vs. HC for both conditions
Toosizadeh et al., 2016 [41] Alterations in gait parameters with peripheral artery disease: The importance of pre-frailty as a confounding variable USA	Observational PAD *n* = 17 (10 men; 7 women) Age (years) = 74 ± 8 BMI (kg/m^2^) = 26.8 ± 3.5 ABI = 0.83 ± 0.04 HC *n* = 24 (12 men; 12 women) Age (years) = 76 ± 7 BMI (kg/m^2^) = 27.9 ± 5.7	Gait 25 steps Two conditions Habitual pace Fast pace	IMU (BioSensics, Newton, MA, USA) *n* = 5 Shin (*n* = 2; right and left) Thigh (*n* = 2; right and left) Lower back (*n* = 1) Frequency not reported	Gait initiation steps Gait initiation distance Gait speed Stride length Gait cycle time Double support Gait speed variability Trunk sway Knee RoM	↑ Gait initiation steps, gait-initiation distance, and trunk sway in PAD vs. HC for both paces ↑ Gait speed in PAD vs. HC for both paces ↓ Stride length, ↑ Gait cycle time and double support in PAD vs. HC for habitual pace ↑ Knee RoM and gait-speed variability in PAD vs. HC for fast pace
Thiede et al., 2016 [42] Gait and balance assessments as early indicators of frailty in patients with known peripheral artery disease USA	Observational Pre-frail PAD *n* = 9 (4 men; 5 women) Age (years) = 74.4 ± 7.5 BMI (kg/m^2^) = 27.1 ± 3.1 ABI = 0.79 ± 0.14 Non-frail PAD *n* = 8 (6 men; 2 women) Age (years) = 73.4 ± 9.9 BMI (kg/m^2^) = 26.4 ± 4.1 ABI = 0.88 ± 0.12 Note: Fried criteria for frailty measurement	Gait 25 steps Three conditions Habitual pace Dual task Fast pace Quiet standing Two conditions Eyes open Eyes closed	IMU (BioSensics, Newton, MA, USA) *n* = 5 Shin (*n* = 2; right and left) Thigh (*n* = 2; right and left) Lower back (*n* = 1) Frequency not reported	Gait Gait speed Stride length Gait cycle time Double support Trunk sway Gait-speed variability Quiet standing CoM sway Ankle sway Hip sway	↓ Gait speed, ↑ Gait cycle time, double support, gait-speed variability in pre-frail PAD vs. non-frail PAD for dual task walking ↑ Double support and trunk sway in pre-frail PAD vs. non-frail PAD for fast pace No significant difference in quiet standing
Najafi et al., 2017 [43] Using plantar electrical stimulation to improve postural balance and plantar sensation among patients with diabetic peripheral neuropathy: A randomized double blinded study USA and Qatar	Interventional (RCT) IG (Wearable plantar electrical stimulation; 1 h daily; 6 weeks; at home) DPN *n* = 17 (12 men; 5 women) Age (years) = 56 ± 11 BMI (kg/m^2^) = 28.7 ± 5.9 HbA1c (%) = 8.8 ± 1.9 Disease duration not reported VPT (Volts) = 41 ± 7 CG (Sham stimulation) DPN *n* = 11 (9 men; 2 women) Age (years) = 64 ± 10 BMI (kg/m^2^) = 31.5 ± 8.0 HbA1c (%) = 9.6 ± 2.2 Disease duration not reported VPT (Volts) = 40 ± 10	Gait 10 m Two conditions Habitual pace Fast pace Quiet standing Two conditions Eyes open Eyes closed	IMU (BioSensics, Newton, MA, USA) *n* = 2 (gait) Shin (*n* = 2; right and left) *n* = 2 (quiet standing) Shin (*n* = 1) lower back (*n* = 1) Frequency not reported	Gait Gait speed Cadence Stride length Stride time Quiet standing CoM sway Ankle sway Hip sway Time points: Baseline 6-week	All variables were similar between IG and CG at baseline ↑ Gait speed, cadence, and stride length ↓ stride time at 6-week vs. baseline in IG ↓ Ankle sway at 6-week vs. baseline in IG
Esser et al., 2018 [44] Single sensor gait analysis to detect diabetic peripheral neuropathy: A proof of principle study UK	Observational DPN *n* = 17 (14 men; 3 women) Age (years) = 63 ± 9 BMI (kg/m^2^) = 33.6 ± 7.6 Duration of DM (years) = 24 ± 13 HbA1c (%) = 8.8 ± 1.0 HC *n* = 42 (30 men; 12 women) Age (years) = 61 ± 4 BMI (kg/m^2^) = 31.6 ± 3.9	Gait 10 m Habitual pace	IMU (NA) *n* = 1 Lower back (*n* = 1) Frequency = 100 Hz	Gait speed Cadence Stride length Stride time	↓ Gait speed, cadence, and stride length ↑ stride time in DPN vs. HC
Kang et al., 2019 [45] The effect of daily use of plantar mechanical stimulation through micro-mobile foot compression device installed in shoe insoles on vibration perception, gait, and balance in people with diabetic peripheral neuropathy USA	Interventional: Micro-mobile foot compression; 4 h daily; 4 weeks Severe DPN *n* = 30 (11 men; 19 women) Age (years) = 68.1 ± 9.7 BMI (kg/m^2^) = 33.4 ± 6.1 Disease duration not reported Blood sugar level not reported VPT (Volts) = 27.4 ± 12.6	Gait 10 m Three conditions Habitual pace Dual task Fast pace Quiet standing Four conditions (two eyes conditions × two foot conditions) Eyes open Eyes closed Double stance Semi-tandem stance	IMU (BioSensics, Newton, MA, USA) *n* = 5 (gait) Shins (*n* = 2; right and left) Thigh (*n* = 2; right and left) Lower back (*n* = 1) Frequency not reported *n* = 2 (quiet standing) Shin (*n* = 1) lower back (*n* = 1) Frequency not reported	Gait Gait speed Stride length Stride time Double support Quiet standing CoM sway Ankle sway Hip sway Time points: Baseline 4-week	↑ Gait speed and stride length at 4-week vs. baseline for habitual pace ↑ Gait speed and stride length ↓ stride time and double support at 4-week vs. baseline for dual task walking ↑ Gait speed ↓ double support at 4-week vs. baseline for fast pace ↓ CoM sway at 4-week vs. baseline for double stance eyes open and eyes closed
Kang et al., 2020 [46] Characteristics of the gait initiation phase in older adults with diabetic peripheral neuropathy compared to control older adults USA	Observational DPN *n* = 38 (20 men; 18 women) Age (years) = 72.6 ± 5.6 BMI (kg/m^2^) = 31.63 ± 6.07 Disease duration not reported Blood sugar level not reported VPT (Volts) = 32 V ± 14 HC *n* = 33 (13 men; 20 women) Age (years) = 77.9 ± 8.2 BMI (kg/m^2^) = 27.05 ± 4.23	Gait 12 m Two conditions Habitual pace Dual task	IMU (BioSensics, Newton, MA, USA) *n* = 5 Shins (*n* = 2; right and left) Thigh (*n* = 2; right and left) Lower back (*n* = 1) Frequency = 100 Hz	Gait-initiation steps Gait-initiation distance Gait speed CoM sway	↑ Gait-initiation steps, gait-initiation distance, and CoM sway, ↓ gait speed in DPN vs. HC for both walking
Ling et al., 2020 [47] The impact of diabetic foot ulcers and unilateral offloading footwear on gait in people with diabetes USA	Observational DPN with DFU wearing unilateral offloading *n* = 12 (10 men; 2 women) Age (years) = 55.6 ± 2.7 BMI (kg/m^2^) = 30.9 ± 1.3 Blood sugar level not reported Disease duration not reported VPT not reported DPN without DFU *n* = 27 (20 men; 7 women) Age (years) = 64.3 ± 1.5 BMI (kg/m^2^) = 30.9 ± 1.0 Blood sugar level not reported Disease duration not reported VPT not reported HC *n* = 47 (22 men; 25 women) Age (years) = 62.9 ± 2.3 BMI (kg/m^2^) = 29.0 ± 0.9	Gait 10 m Habitual pace	IMU (BioSensics, Newton, MA, USA) *n* = 5 Shins (*n* = 2; right and left) Thigh (*n* = 2; right and left) Lower back (*n* = 1) Frequency not reported	Gait speed Stride length Gait cycle time Double support Gait-speed variability Stride-length variability Double-support limp Step-length limp	↓ Gait speed, and stride length, ↑ gait cycle time, double-support limp, and step- length limp in DPN with DFU wearing unilateral offloading vs. DPN without DFU and HC ↑ Double support, gait-speed variability, stride-length variability in DPN with DFU wearing unilateral offloading and DPN without DFU vs. HC
Du et al., 2021 [48] The feasibility and effectiveness of wearable sensor technology in the management of elderly diabetics with foot ulcer remission: A proof-of-concept pilot study with six cases China	Observational Longitudinal DM with recently recovered from DFU *n* = 6 (sex ratio not reported) Offloading footwear group (*n* = 3) Regular footwear group (*n* = 3) Age (years): between 55–80 Duration of DM: lasting for > 5 years Blood sugar level not reported VPT not reported	Gait 1 min Habitual walk Quiet standing Four conditions (two eyes conditions × two surface conditions) Eyes open Eyes closed Hard surface Soft surface	IMU (BioSensics, Newton, MA, USA) *n* = 5 (gait) Shin (*n* = 2; right and left) Thigh (*n* = 2; right and left) Lower back (*n* = 1) Frequency = 100 Hz *n* = 2 (quiet standing) Shin (*n* = 1) lower back (*n* = 1) Frequency = 100 Hz	Gait: Gait speed Stride length Double support Swing phase Quiet standing: CoM sway Ankle sway Hip sway Timepoints: Baseline 1-week 1-month 4-month 6-month	↑ Gait speed and stride length, ↓ double support in offloading footwear group Quiet standing remained similar
Lanzi et al., 2021 [49] Supervised exercise training improves 6 min walking distance and modifies gait pattern during pain-free walking condition in patients with symptomatic lower extremity peripheral artery disease Switzerland	Interventional: Supervised exercise training PAD *n* = 29 (15 men; 14 women) Age (years) = 65.4 ± 9.9 BMI (kg/m^2^) = 28.7 ± 6.2 ABI = 0.79 ± 0.14	Gait 6 min walk test Habitual pace	IMU (GaitUp, Renens, Switzerland) *n* = 2 Sensor placement not specified Frequency not reported	Gait speed Stride length Stride time Stride frequency Double support Stance phase Swing phase Loading response Heel-strike pitch angle Toe-off pitch angle Max-heel clearance First max-toe clearance Second max-toe clearance Minimum toe clearance Time points: Baseline 3-month	↑ Gait speed, stride length, swing phase, and loading response, ↓ stance phase at 3-month vs. baseline ↑ Toe off pitch angle at 3-month vs. baseline

Abbreviations: DM = Diabetes mellitus; DPN = Diabetic peripheral neuropathy; DFU = Diabetic foot ulcer; BMI = Body-mass index; HC = Healthy controls; RCT = Randomized controlled trial; IG = Intervention group; CG = Control group; IMU = Inertial measurement unit; CoM = Center of mass; DFO = Dynamic foot orthoses; PAD = Peripheral artery disease; ABI = Ankle brachial index; RCW = Removable case walker; RoM = Range of motion; NA = Not available; VPT = Vibration perception threshold.

## Data Availability

No data were generated.
